# The effects of fetal or neonatal hypoxia and genetic variants on age at onset of schizophrenia

**DOI:** 10.1192/j.eurpsy.2022.534

**Published:** 2022-09-01

**Authors:** V. Golimbet, T. Lezheiko, M. Gabaeva, G. Korovaitseva, N. Kolesina, V. Plakunova

**Affiliations:** Mental Health Research Center, Clinical Genetics Laboratory, Moscow, Russian Federation

**Keywords:** gene-environmental interaction, schizophrénia, hypoxia, age at disease onset

## Abstract

**Introduction:**

Fetal or neonatal hypoxia (FoNH) is a known risk factor for schizophrenia. It has been hypothesized that FoNH induced expression of schizophrenia susceptibility genes (Schmidt-Kastner et al. 2012, Giannopoulou et al. 2018).

**Objectives:**

To test this hypothesis, we explore the effects of FoNH and some genetic variants on age at onset (AAO) of schizophrenia.

**Methods:**

The study included 1670 patients (women 1021 (61.1%), mean age 34.6 (SD 13.6), mean age at disease onset 25.4 (10.5) years) with ICD-10 diagnosis of schizophrenia or schizoaffective psychosis. The effects of FoNH in interaction with sex, family history (FH) and genetic variants on AAO of schizophrenia were evaluated. Polymorphisms rs2514218 DRD2 (n=943), Val66Met BDNF (n=820) and VNTR AS3MT (n=804) were genotyped.

**Results:**

Among all patients studied 179 (10.8%) had experienced FoNH. Regression model showed that FoNH, sex and FH of schizophrenia contribute significantly (p=0.000) to AAO. In the FoNH group, AAO was lower compared to the group without FoNH (20.7 (6.2) vs 25.5 (10.) years). When comparing men and women, there was a difference between FoNH and non- FoNH subgroups only in women (p=0.000). No interaction between FH and FoNH was observed though positive FH had an effect on AAO. There was the interaction effect of VNTR AS3MT and FoNH on AAO. In the FoNH group, carriers of 2 repeats had younger AAO compared to the carriers homozygous for 3 repeat variant (19.6 (4.9) vs 22. (7.6) years).

**Conclusions:**

We demonstrate the interaction effects of FoNH and VNTR AS3MT polymorphism on AAO of schizophrenia.

**Disclosure:**

No significant relationships.

